# Supplementary material for: Dietary Outcomes of the ‘Healthy Youngsters, Healthy Dads’ Randomised Controlled Trial
Source: Nutrients. 2021 Sep 22;13(10):3306. doi: 10.3390/nu13103306 (PMC8541030; doi:10.3390/nu13103306)
Supplement: Supplementary file 1 [file nutrients-13-03306-s001.zip › nutrients-1366718-supplementary.pdf]

Supplementary Material

Dietary outcomes of the 'Healthy Youngsters, Healthy Dads' Randomized Controlled Trial

Lee M Ashton, Philip J Morgan*, Jacqueline Grounds, Myles D Young, Anna T Rayward, Alyce T. Barnes, Emma R. Pollock, Stevie-Lee Kennedy, Kristen L. Saunders Clare E Collins.

*Correspondence: Philip.morgan@newcastle.edu.au; Tel.: +61-2-49-217265

<u>**Contents**</u>

Supporting information item	Page
**Table S1.** Description of intervention components in the 'Healthy Youngsters, Healthy Dads' program	2
**Table S2.** Change in dietary intake of children enrolled in the Healthy Youngsters Healthy Dads (HYHD) RCT (Completers) n=82	4
**Table S3.** Change in dietary intake of fathers enrolled in the Healthy Youngsters Healthy Dads (HYHD) RCT (completers) n=79	7
**Table S4**. Change in dietary intake of children enrolled in the Healthy Youngsters Healthy Dads (HYHD) RCT (per-protocol) n=91	10
**Table S5:** Change in dietary intake of fathers enrolled in the Healthy Youngsters Healthy Dads (HYHD) RCT (per-protocol) n=91	13

Table S1. Description of intervention components in the 'Healthy Youngsters, Healthy Dads' program

Intervention component	Overview	Detail	Behaviour change techniques	Targeted theoretical mediators
'Fathers-only' workshop	• 2 x 2hours delivered by a trained facilitator with physical education qualifications.<br>• Held on the Thursday evenings before and after the first father-and-child session.	Topics covered:<br>• Optimising health in the early years<br>• The unique and powerful influence of fathers<br>• Positive parenting strategies<br>• SMART goal setting<br>• Physical activity parenting<br>• Fundamental movement skills<br>• Healthy eating for families<br>• Screen-time parenting	• Social support (practical, emotional)<br>• Increase positive emotions<br>• Instructions on how to perform the behaviour<br>• Information about consequences (health, social and environmental, emotional)<br>• Demonstration of the behaviour<br>• Graded tasks<br>• Credible source<br>• Identification of self as role model<br>• Framing/reframing<br>• Verbal persuasion about capability	• Social support/ relatedness (SCT/SDT)<br>• Autonomy (SDT)<br>• Self-efficacy/ perceived competence (SCT/SDT)<br>• Outcome expectations (SCT)<br>• Goals (SCT)
Weekly group sessions for fathers and children	• 8 x weekly 75-minute sessions (20-minute *Education session* plus 55-minute *Practical session).*<br>• Mothers/partners and siblings invited to attend week 5 session.<br>• Four facilitators were trained to deliver the program, and each session was delivered by two facilitators. All facilitators had education qualifications.<br>• Participants were offered one of three Saturday morning timeslots, delivered by two facilitators. Some facilitators delivered more than one session each week.	*Education session:*<br>• Icebreakers and overview of weekly focus which alternated between physical activity (e.g. rough and tumble play, sport skills) and healthy eating (e.g. vegetables, fruit).<br>• Each week introduced a new animal character and a slogan.<br>*Practical session:*<br>• Designed to increase preschool-aged children's motivation and skills to engage in physical activity fun and active father-child games.<br>• Each session targeted rough and tumble play (15 min), sport skills (i.e., FMS) (15 min), and aerobic and muscular fitness (15 min). There was a cool-down component (5 mins) at the end of every session and transition time was accounted for (5 mins).<br>• Seven sports skills targeted (2/week): catch, kick, one-handed strike, two-handed strike, bounce, overhand throw and underhand throw.		

Home-based program	• Activity Handbook containing a range of engaging activities, challenges and sport skills.<br>• One Yamax SW200 *pedometer* to assist with monitoring step counts.<br>• Sticker chart to earn the animal sticker of the week (e.g. Charlie the Chimpanzee) for one home challenge and bonus stickers for completing more than one activity (e.g. apple, basketball).<br>• One home challenge task was minimum requirement.	Fathers asked to use Activity Handbook each week and record completed home tasks:<br>• Home challenges based on weekly theme (e.g., sock wrestle)<br>• Sport skill games (e.g., capture the target)<br>• Step count monitoring (using pedometer)<br>• Co-physical activity (≥ 10-minute bouts)<br>• SMART goals relating to physical activity, healthy eating, screen time and parenting<br>• Review SMART goal progress<br>• Dad task (e.g. bring at least one piece of fruit with you to work every day this week.)	• Material incentive<br>• Instructions on how to perform the behaviour<br>• Graded tasks<br>• Prompts/cues<br>• Increase positive emotions<br>• Goal setting<br>• Action planning<br>• Self-monitoring	• Goals (SCT)<br>• Social support/ relatedness (SCT/SDT)<br>• Autonomy<br>• Self-efficacy /perceived competence (SCT/ SDT)

Abbreviations: SCT = social cognitive theory; SDT = self-determination theory; FMS= fundamental movement skills; SMART = Specific, Measurable, Achievable, Relevant, Timely

Table S2: Change in dietary intake of children enrolled in the Healthy Youngsters Healthy Dads (HYHD) RCT (Completers) n=82

			Baseline	10 weeks change from baseline (Mean, 95% CI)			9 months change from baseline (Mean, 95% CI)		
**Outcome**	**NRV [1-4]**	**Group**	**Mean (SE)**	**Within group[a]**	**Mean difference between groups[b]**	***p*-value [Cohen's *d*]**	**Within group[c]**	**Mean difference between groups[b]**	***p*-value [Cohen's *d*]**
Energy (kJ/day)[*d,e]	-	Intervention	6013 (360)	-469 (-1033, 94)	-285 (-1113, 543)	.495 [0.15]	+509 (-326, 1344)	**-946 (-2172, 281)**	**.013 [0.34]**
		Control	5815 (387)	-184 (-790, 422)			+1454 (556, 2353)		
Diet Quality Score (max score is 73, higher score = better diet quality)									
Total ARFS	-	Intervention	31.2 (1.4)	+0.9 (-0.7, 2.4)	+0.4 (-1.9, 2.7)	.737 [0.07]	+0.6 (0.9, 2.2)	+0.7 (-1.6, 3.0)	.548 [0.13]
		Control	33.4 (1.5)	+0.5 (-1.2, 2.2)			-0.1 (-1.7, 1.6)		
Macronutrients (% of total energy intake or g/day)									
Protein (%)	AMDR: 15-25%	Intervention	16.6 (0.3)	+0.2 (-0.3, 0.7)	+0.4 (-0.4, 1.1)	.340 [0.21]	+0.2 (-0.3, 0.7)	+0.5 (-0.3, 1.2)	.197 [0.29]
		Control	16.8 (0.3)	-0.2 (-0.7, 0.4)			-0.3 (-0.8, 0.3)		
Carbohydrate (%)	AMDR: 45-65%	Intervention	46.9 (0.8)	0.0 (-1.1, 1.1)	-0.9 (-2.4, 0.7)	.274 [0.24]	+0.7 (-0.6, 2.0)	-0.8 (-2.7, 1.1)	.397 [0.19]
		Control	46.8 (0.9)	+0.9 (-0.3, 2.0)			+1.5 (0.1, 2.9)		
Total sugars (g/day)[d]	-	Intervention	91.0 (5.6)	-8.6 (-19.2, 2.0)	-7.6 (-23.1, 8.0)	.340 [0.21]	4.2 (-6.4, 14.8)	-9.4(-24.9, 6.2)	.237 [0.26]
		Control	86.0 (6.0)	-1.1 (-12.5, 10.3)			13.6 (2.2, 25.0)		
Fat (%)	AMDR: 20-35%	Intervention	37.0(0.7)	-0.5 (-1.4, 0.4)	+0.5 (-0.8, 1.87)	.439 [0.17]	**-1.3 (-2.5, -0.1)**	+0.3 (-1.4, 2.0)	.720 [0.08]
		Control	36.8 (0.7)	-1.0 (-2.0, -0.4)			**-1.6 (-2.9, -0.4)**		
Sat fat (%)[d]	<10%	Intervention	16.4 (0.4)	-0.5 (-1.2, 0.2)	+0.2 (-0.7, 1.2)	.636[0.11]	-1.0 (-1.7, -0.4)	-0.1 (-1.1, 0.9)	.844 [0.04]
		Control	16.0 (0.4)	-0.7 (-1.5, 0.0)			-0.9 (-0.9, 0.5)		
Micronutrients									
Fibre (g/day)[d]	AI: 14g (aged 3yrs), 18g (aged 4-5 yrs)	Intervention	17.1 (1.1)	-0.4 (-2.2, 1.4)		.602 [0.12]	+3.3 (0.5, 6.0)		
		Control	17.4 (1.2)	+0.3 (-1.6, 2.2)	-0.7 (-3.3, 2.0)		+5.8 (2.8, 8.7)	-2.5 (-6.5, 1.51)	.218 [0.27]
Sodium (mg/day)[d]	AI: 200-400mg (aged 3yrs), 300-600 (aged 4-5yrs)	Intervention	1290.8 (79.6)	**-123.7 (-245.2, -2.3)**		.257 [0.25]	+109.7 (-72.8, 292.2)		
		Control	1222.6 (85.7)	-21.3 (-152.0, 109.382)	-102.4 (-280.8, 76.0)		+399.8 (203.4, 596.2)	**-290.1 (-558.3, -21.9)**	**.034 [0.48]**
Healthy, nutrient dense core foods (% of total energy intake)									
Core foods	≥85%	Intervention	71.5 (1.6)	**+3.7 (1.6, 5.7)**	+2.9 (-0.1, 5.9)	.056 [0.43]	+1.8 (-0.3, 3.8)	+3.0 (0.0, 5.9)	.053 [0.43]
		Control	72.7 (1.7)	+0.7 (-1.5, 2.9)			-1.2 (-3.4, 1.0)		

Vegetables [f]	-	Intervention	6.5 (0.7)	+0.6 (-0.4, 1.6)	+0.5 (-0.9, 2.0)	.486 [0.15]	+0.1 (-0.9, 1.1)	+0.4 (-1.1, 1.8)	.607[0.11]
		Control	7.7 (0.7)	-0.1 (-1.0, 1.1)			-0.3 (-1.4, 0.8)		
Fruit	-	Intervention	10.1 (0.7)	**+1.5 (0.1, 2.8)**	-0.2 (-2.1, 1.8)	.878 [0.03]	+1.3 (-0.1, 2.6)	+0.8 (-1.1, 2.8)	.398 [0.19]
		Control	9.8 (0.8)	**+1.6 (0.2, 3.0)**			+0.4 (-1.0, 1.8)		
Meats [d]	-	Intervention	7.9 (0.7)	+0.6 (-0.6, 1.9)	+0.1 (-1.8, 2.0)	.907 [0.03]	+0.7 (-0.7, 2.0)	+0.5 (-1.3, 2.4)	.582 [0.12]
		Control	8.2 (0.7)	+0.5 (-0.8, 1.9)			+0.2 (-1.2, 1.5)		
Vegetarian protein sources	-	Intervention	2.9 (0.4)	+0.1 (-0.5, 0.8)		.567 [0.13]	+0.2 (-0.5, 0.8)		
		Control	3.0 (0.5)	+0.1 (-0.8, 0.5)	+0.3 (-0.7, 1.2)		-0.1 (-0.8, 0.5)	+0.3 (-0.6, 1.2)	.534 [0.14]
Grains [d]	-	Intervention	18.6 (1.0)	+1.2 (-0.8, 3.3)	+0.8 (-2.2, 3.7)	.620 [0.11]	+2.3 (0.3, 4.4)	-0.1 (-3.1, 2.9)	.946 [0.01]
		Control	20.0 (1.1)	+0.5 (-1.7, 2.7)			+2.4 (0.2, 4.6)		
Breakfast cereals	-	Intervention	9.0 (0.8)	+0.6 (-1.0, 2.2)	+0.4 (-2.0, 2.7)	.768 [0.07]	+1.0 (-0.6, 2.6)	+1.3 (-1.1, 3.7)	.284 [0.24]
		Control	9.1 (0.9)	+0.2 (-1.5, 2.0)			-0.3 (-2.0, 1.5)		
Dairy [d]	-	Intervention	27.8 (1.5)	-0.7 (-3.4, 2.0)	+1.1 (-2.9, 5.1)	.584 [0.11]	-3.3 (-6.0, -0.6)	+0.4 (-3.6, 4.4)	.829 [0.05]
		Control	26.3 (1.6)	-1.8 (-4.7, 1.1)			-3.7 (-6.6, -0.8)		
Energy-dense nutrient-poor (EDNP) foods (% of total energy intake)									
EDNP foods	<1 serve (~5-20%)	Intervention	28.5 (1.6)	**-3.7 (-5.7, -1.6)**	-2.9 (-5.9, -0.1)	.056 [0.43]	-1.8 (-3.8, 0.3)	-3.0 (-5.9, -0.0)	.053 [0.43]
		Control	27.3 (1.7)	-0.7 (-2.9, 1.5)			+1.2 (-1.0, 3.4)		
Sugar-sweetened beverages	-	Intervention	0.9 (0.3)	-0.1 (-0.5, 0.3)		.785 [0.06]	-0.1 (-0.5, 0.3)		
		Control	0.7 (0.3)	-0.0 (-0.5, 0.3)	-0.1 (-0.7, 0.5)		-0.1 (-0.5, 0.4)	-0.0 (-0.6, 0.6)	.968 [0.01]
Prepacked snacks	-	Intervention	3.7 (0.5)	**-1.4 (-2.2, -0.6)**	**-1.6 (-2.8, -0.4)**	**.009 [0.59]**	**-1.4 (-2.2, -0.6)**	**-2.3 (-3.4, -1.1)**	**.000 [0.83]**
		Control	3.0 (0.5)	-0.2 (-0.7, 1.1)			**+0.9 (0.0, 1.8)**		
Confectionary	-	Intervention	4.9 (0.5)	**-1.0 (-1.9, -0.1)**	-0.3 (-1.6, 1.0)	.635 [0.11]	-0.3 (-1.2, 0.5)	+0.3 (-1.0, 1.6)	.630 [0.11]
		Control	5.2 (0.6)	-0.7 (-1.7, 0.3)			-0.7 (-0.9, 0.3)		
Baked products	-	Intervention	5.3 (0.5)	**-0.9 (-1.8, 0.0)**	-0.7 (-2.0, 0.6)	.279 [0.24]	-0.7 (-1.5, 0.2)	-0.6 (-1.9, 0.7)	.350 [0.21]
		Control	5.1 (0.5)	-0.2 (-1.1, 0.7)			-0.1 (-1.0, 0.9)		
Takeaway/ fast foods [d]	-	Intervention	6.4 (0.6)	0.0 (-0.9, 0.8)	-0.5 (-1.7, 0.8)	.459 [0.16]	**+1.0 (-0.2, 1.9)**	-0.7 (-1.9, +0.6)	.294 [0.23]
		Control	6.0 (0.6)	+0.4 (-0.5, 1.3)			+1.7 (+0.8, 2.6)		
Condiments	-	Intervention	3.1 (0.3)	+0.1 (-0.6, 0.8)	0.00 (-1.0, 1.0)	.981 [0.01]	-0.3 (-0.9, 0.4)	-0.1 (-1.1, 0.9)	.851 [0.04]
		Control	2.8 (0.3)	+0.1 (-0.6, 0.8)			-0.2 (-0.9, 0.6)		
Fatty meats	-	Intervention	1.8 (0.2)	0.0 (-0.4, 0.5)	0.0 (-0.6, 0.7)	.945 [0.02]	+0.2 (-0.2, 0.6)	+0.3 (-0.4, 0.9)	.432 [0.17]
		Control	1.7 (0.3)	0.0 (-0.5, 0.5)			-0.1 (-0.5, 0.4)		
Meals *(% of total energy intake)*									

Meals with vegetables [g]	-	Intervention	5.2 (0.6)	+0.6 (-0.4, 1.7)	+0.1 (-1.4, 1.7)	.886 [0.03]	0.0 (-1.1, 1.0)	-0.0 (-1.6, 1.5)	.951 [0.01]
		Control	5.1 (0.6)	+0.5 (-0.6, 1.6)			0.0 (-1.1, 1.2)		
Meals without vegetables [h]	-	Intervention	1.6 (0.3)	-0.2 (-0.8, 0.4)	+0.1 (-0.9, 1.0)	.906 [0.03]	+0.3 (-0.3, 0.9)	+0.5 (-0.4, 1.4)	.256 [0.25]
		Control	1.7 (0.3)	-0.2 (-0.9, 0.4)			-0.2 (-0.9, 0.5)		

Bold denotes a significant difference. *1 kcal = 4.186 kJ.a. 10 week value minus baseline; b. Within-group difference (intervention) minus within-group difference (control); c. 9 month value minus baseline; d. Adjusted for child's age; e. Truncated to account for outliers [5] (>3.29 SD truncated to next highest value plus 1); f. Adjusted for child's sex; g. composite score of all protein meals that were consumed with vegetables, h. composite score of all protein meals that were consumed without vegetables; ARFS; Australian Reccommended Food Score, NRV; Nutrient Reference Value, AMDR; Acceptable Macronutrient Distribution Ranges, AI; Acceptable Intake

Table S3: Change in dietary intake of fathers enrolled in the Healthy Youngsters Healthy Dads (HYHD) RCT (completers) n=79

			**Baseline**	**10 weeks change from baseline (Mean, 95% CI)**			**9 months change from baseline (Mean, 95% CI)**		
**Outcome**	**NRV [2-4,6]**	**Group**	**Mean (SE)**	**Within group**	**Mean difference between groups**	***p*-value [Cohen's *d*]**	**Within group**	**Mean difference between groups**	***p*-value [Cohen's *d*]**
Energy (kJ day)*[d,e]	-	Intervention	10512 (510)	**-1092 (-1770, 413)**	-912 (-1890, +66)	.067 [0.41]	**-701 (-1379, -22)**	-892 (-1869, +86)	.074 [0.41]
		Control	10780 (530)	-180 (-884, 525)			+191 (-514, +895)		
Diet Quality Score (max score is 73, higher score = better diet quality)									
Total ARFS	-	Intervention	32.9 (1.4)	+0.9 (-1.1, 2.9)	+1.2 (-1.7, 4.1)	.415 [0.18]	+0.7 (-1.3, 2.7)	+2.0 (-0.9, 4.9)	.178 [0.30]
		Control	34.1 (1.4)	-0.3 (-2.4, 1.8)			-1.3 (-3.4, 0.8)		
Macronutrients (% of total energy intake)									
Protein	AMDR: 15-25%	Intervention	17.1 (0.5)	+0.7 (-0.2, 1.6)	+0.8 (-0.5, 2.0)	.217 [0.28]	+0.7 (-0.2, 1.5)	+0.6 (-0.6, 1.9)	.320 [0.22]
		Control	17.2 (0.5)	-0.1 (-1.0, 0.8)			0.0 (-0.9, 0.9)		
Carbohydrate	AMDR: 45-65%	Intervention	43.7 (1.1)	+0.6 (-1.0, 2.3)	-0.1 (-2.4, 2.3)	.967 [0.01]	-0.0 (-1.7, 1.7)	+0.3 (-2.1, 2.7)	.778 [0.06]
		Control	44.0 (1.1)	+0.7 (-1.0, 2.4)			-0.4 (-2.1, 1.4)		
Fat	AMDR: 20-35%	Intervention	36.4 (0.9)	-0.9 (-2.3, 0.5)	-0.3 (-2.3, 1.7)	.786 [0.06]	-0.6 (-2.0, 0.8)	-0.5 (-2.5, 1.5)	.615 [0.11]
		Control	36.6 (0.9)	-0.6 (-2.0, 0.8)			-0.1 (-1.5, 1.3)		
Saturated fat	<10%	Intervention	13.4 (0.4)	-0.5 (-1.3, 0.2)	-0.4 (-1.5, 0.7)	.454 [0.17]	-0.4 (-1.1, 0.3)	-0.7 (-1.8, 0.3)	.177 [0.31]
		Control	13.8 (0.4)	+0.1 (-0.9, 0.6)			+0.3 (-0.4, 1.1)		
Alcohol	-	Intervention	3.0 (0.5)	**-0.8 (-1.4, -0.3)**	-0.8 (-1.6, 0.1)	.073 [0.41]	-0.2 (-1.5, 1.1)	-0.8 (-2.7, 1.1)	.389 [0.45]
		Control	2.3 (0.5)	-0.1 (-0.7, 0.5)			+0.6 (-0.7, 2.0)		
Micronutrients									
Fibre (g/day)[d]	AI: 30g/day (male aged 19-70y)	Intervention	31.6 (1.6)	-0.1 (-2.0, 1.9)	-0.5 (-3.3, 2.2)	.703 [0.09]	-0.0 (-3.9, 3.8)	-1.4 (-7.0, 4.2)	.617 [0.11]
		Control	32.8 (1.7)	+0.5 (-1.5, 2.5)			+1.4 (-2.7, 5.4)		
Sodium (mg/day)	SDT: <2000mg/day (male aged 19-70y).	Intervention	2257 (125)	**-316 (-469, -165)**	**-266 (-485, -46.9)**	**.018 [0.54]**	-205 (-683, 274)	-416 (-1105, 274)	.234 [0.27]
		Control	2369 (129)	-51 (-208, 107)			+211 (-286, 708)		
Healthy, nutrient dense core foods (% of total energy intake)									
Core foods	≥85%	Intervention	60.5 (1.8)	**+5.8 (3.1, 8.5)**	**+3.91 (0.0, 7.8)**		**+4.2 (1.6, 6.9)**	+3.1 (-0.8, 6.9)	.120 [0.35]

		Control	61.7 (1.9)	+1.9 (-0.9, 4.7)		**.047 [0.45]**	+1.2 (-1.6, 4.0)		
Vegetables	-	Intervention	8.8 (0.7)	**+1.1 (0.1, 2.1)**	+0.6 (-0.8, 2.1)	.402 [0.19]	+0.8 (-0.2, 1.8)	+0.5 (-0.9, 2.0)	.468 [0.16]
		Control	7.9 (0.7)	+0.5 (-0.6, 1.5)			+0.3 (-0.8, 1.3)		
Fruit	-	Intervention	6.5 (0.7)	**+1.7 (0.5, 2.9)**	+1.4 (0.3, 3.1)	.116 [0.36]	+0.2 (-1.0, 1.4)	+0.3 (-1.4, 2.0)	.730 [0.08]
		Control	6.2 (0.7)	+0.4 (-0.9, 1.6)			-0.1 (-1.3, 1.2)		
Meats	-	Intervention	13.6 (1.1)	+1.4 (-0.8, 3.5)	+2.0 (-1.1, 5.1)	.204 [0.29]	+0.6 (-1.5, 2.8)	+1.3 (-1.8, 4.4)	.420 [0.18]
		Control	13.4 (1.1)	-0.6 (-2.8, 1.6)			-0.6 (-2.9, 1.6)		
Vegetarian protein sources	-	Intervention	4.2 (0.5)	-0.2 (-1.0, 0.5)	-0.1 (-1.2, 1.0)	.875 [0.04]	-0.5 (-1.3, 0.2)	-0.7 (-1.8, 0.4)	.215 [0.28]
		Control	3.4 (0.5)	-0.1 (-0.9, 0.7)			+0.2 (-0.5, 1.1)		
Grains	-	Intervention	19.0 (1.3)	+1.3 (-0.9, 3.5)	+0.3 (-2.9, 3.5)	.843 [0.04]	**+3.2 (1.0, 5.4)**	**+4.4 (1.2, 7.6)**	**.007 [0.62]**
		Control	21.4 (1.3)	+1.0 (-1.3, 3.3)			-1.2 (-3.5, 1.1)		
Breakfast cereals	-	Intervention	5.4 (0.8)	+0.5 (-0.6, 1.7)	+0.3 (-1.9, 1.4)	.764 [0.07]	+1.0 (-0.2, 2.1)	+1.5 (-0.1, 3.2)	.069 [0.41]
		Control	6.7 (0.8)	+0.8 (-0.4, 2.0)			-0.6 (-1.7, 0.6)		
Dairy	-	Intervention	8.2 (1.0)	+0.3 (-1.8, 2.3)	-0.6 (-3.6, 2.3)	.676 [0.09]	0.0 (-2.1, 2.0)	-2.2 (-5.2, 0.7)	.138 [0.34]
		Control	9.8 (1.0)	+0.9 (-1.2, 3.0)			**+2.2 (0.1, 4.3)**		
Energy-dense nutrient-poor (EDNP) foods (% of total energy intake)									
EDNP foods	<3 serves (~5-20%)	Intervention	39.5 (1.8)	**-5.8 (-8.5, -3.1)**		**.047 [0.45]**	**-4.2 (-6.9, -1.6)**	-3.1 (-6.9, 0.8)	.120 [0.35]
		Control	38.0 (1.9)	-1.9 (-4.7, 0.9)	**-3.9 (-7.8, 0.0)**		-1.2 (-4.0, 1.6)		
Sugar-sweetened beverages	-	Intervention	2.4 (0.5)	0.0 (-0.8, 0.8)	+0.0 (-1.2, 1.2)	.966 [0.01]	**-0.8 (-1.5, -0.1)**	-0.3 (-1.3, 0.6)	.500 [0.15]
		Control	1.5 (0.5)	0.0 (-0.9, 0.9)			-0.5 (-1.2, 0.2)		
Prepacked snacks	-	Intervention	3.5 (0.4)	-0.4 (-1.2, 0.4)	-0.7 (-1.8, 0.4)	.219 [0.28]	-0.1 (-0.9, 0.7)	-0.7 (-1.8, 0.5)	.257 [0.26]
		Control	2.5 (0.4)	+0.3 (-0.5, 1.1)			+0.6 (-0.3, 1.4)		
Confectionary	-	Intervention	6.6 (0.7)	**-2.0 (-3.2, -0.8)**	-1.6 (-3.3, -0.2)	.079 [0.40]	**-1.9 (-3.1, -0.7)**	**-1.7 (-3.5, 0.0)**	**.049 [0.45]**
		Control	6.3 (0.7)	-0.4 (-1.7, 0.8)			-0.2 (-1.4, 1.1)		
Baked products	-	Intervention	4.6 (0.6)	0.0 (-1.0, 0.9)	-0.3 (-1.7, 1.1)	.633 [0.11]	0.0 (-1.0, 0.9)	-0.3 (-1.7, 1.1)	.714 [0.08]
		Control	4.8 (0.6)	+0.3 (-0.7, 1.3)			+0.2 (-0.8, 1.2)		
Takeaway/ fast foods	-	Intervention	10.9 (1.2)	-1.2 (-2.7, 0.4)	+0.4 (-1.9, 2.7)	.721 [0.08]	-1.0 (-2.3, 0.4)	+0.2 (-1.7, 2.1)	.828 [0.05]
		Control	12.1 (1.3)	-1.6 (-3.2, 0.1)			-1.2 (-2.5, 0.2)		
Condiments	-	Intervention	3.5 (0.4)	**-0.8 (-1.5, 0.1)**	-0.5 (-1.6, 0.5)	.303 [0.23]	-0.6 (-1.3, 0.1)	-0.6 (-1.6, 0.5)	.268 [0.25]
		Control	3.4 (0.4)	-0.2 (-1.0, 0.5)			0.0 (-0.7, 0.7)		
Fatty meats	-	Intervention	2.7 (0.3)	-0.1 (-0.6, 0.3)	-0.0 (-0.7, 0.6)	.901 [0.03]	0.0 (-0.4, 0.5)	+0.7 (-0.0, 1.4)	.055 [0.44]
		Control	2.8 (0.3)	-0.1 (-0.6, 0.4)			**-0.6 (-1.1, -0.1)**		

Meals *(% of total energy intake)*									
Meals with vegetables (%)[f]	-	Intervention	8.6 (0.8)	+1.2 (-0.3, 2.7)	+1.6 (-0.6, 3.7)	.155 [0.32]	+0.8 (-0.7, 2.3)	+1.5 (-0.7, 3.6)	.183 [0.30]
		Control	8.6 (0.8)	-0.4 (-2.0, 1.2)			-0.7 (-2.2, 0.9)		
Meals without vegetables (%)[g]	-	Intervention	2.6 (0.6)	-0.2 (-1.3, 0.9)	+0.0 (-1.6, 1.6)	.983 [0.00]	-0.5 (-1.6, 0.6)	-0.2 (-1.8, 1.4)	.829 [0.04]
		Control	2.7 (0.6)	-0.2 (-1.4, 0.9)			-0.3 (-1.5, 0.8)		

Bold denotes a significant difference. *1 kcal = 4.186 kJ.a. 10 week value minus baseline; b. Within-group difference (intervention) minus within-group difference (control); c. 9 month value minus baseline; d. Adjusted for SES, e. Truncated to account for outliers [5] (>3.29 SD truncated to next highest value plus 1) ; f. composite score of all protein meals that were consumed with vegetables, g. composite score of all protein meals that were consumed without vegetables; ARFS; Australian Reccommended Food Score, NRV; Nutirent Reference Value, AMDR; Acceptable Macronutrient Distribution Ranges, AI; Acceptable Intake, SDT: Suggested Dietary Target

Table S4: Change in dietary intake of children enrolled in the Healthy Youngsters Healthy Dads (HYHD) RCT (per-protocol) n=91

			Baseline	10 weeks change from baseline (Mean, 95% CI)			9 months change from baseline (Mean, 95% CI)		
**Outcome**	**NRV [1-4]**	**Group**	**Mean (SE)**	**Within group[a]**	**Mean difference between groups[b]**	***p*-value [Cohen's *d*]**	**Within group[c]**	**Mean difference between groups[b]**	***p*-value [Cohen's *d*]**
Energy (kJ/day)[*d, e]	-	Intervention	6144 (441)	-338 (-1331, 655)	-610 (-1859, 638)	.336 [0.20]	+739 (-254, 1732)	-1151 (-2403, -101)	.071 [0.38]
		Control	5727 (312)	+272 (-484, 1029)			+1890 (1128, 2652)		
Diet Quality Score (max score is 73, higher score = better diet quality)									
Total ARFS	-	Intervention	30.6 (1.7)	+1.8 (-0.1, 3.7)	+1.1 (-1.4, 3.5)	.386 [0.18]	+1.5 (-0.4, 3.4)	+1.2 (-1.3, 3.6)	.348 [0.20]
		Control	34.2 (1.2)	+0.8 (-0.7, 2.2)			0.4 (-1.1, 1.9)		
Macronutrients (% of total energy intake or g/day)									
Protein (%)	AMDR: 15-25%	Intervention	16.1 (0.4)	+0.6 (-0.0, 1.2)	+0.7 (-0.1, 1.5)	.078 [0.37]	+0.3 (-0.3, 0.9)	+0.4 (-0.4, 1.2)	.284 [0.23]
		Control	16.7 (0.3)	-0.1 (-0.6, 0.4)			-0.1 (-0.6, 0.4)		
Carbohydrate (%)	AMDR: 45-65%	Intervention	47.8 (0.9)	-0.5 (-2.0, 0.9)	-1.6 (-3.4, 0.3)	.094 [0.35]	+0.6 (-0.9, 2.1)	-0.8 (-2.7, 1.0)	.374 [0.19]
		Control	46.9 (0.6)	**+1.0 (0.3, 2.6)**			**+1.4 (0.3, 2.6)**		
Total sugars (g/day)[d]		Intervention	94.1 (7.3)	-5.4 (19.0, 8.2)	-10.9 (-28.1, 6.3)	.211 [0.26]	+7.9 (-5.7, 21.5)	-10.9 (-28.1, 6.4)	.214 [0.26]
		Control	85.3 (5.2)	5.6 (-4.9, 16.0)			**+18.8 (8.3, 29.4)**		
Fat (%)	AMDR: 20-35%	Intervention	36.8 (0.8)	-0.2 (-1.3, 0.8)	+1.0 (-0.4, 2.3)	.153 [0.30]	-1.3 (-2.8, 0.2)	+0.3 (-1.6, 2.2)	.766 [0.06]
		Control	36.9 (0.6)	**-1.2 (-2.0, -0.3)**			**-1.6 (-2.7, -0.5)**		
Sat fat (%)	<10%	Intervention	16.3 (0.5)	-0.2 (-1.0, 0.6)	+0.6 (-0.5, 1.6)	.269 [0.23]	**-1.3 (-2.1, -0.5)**	-0.2 (-1.3, 0.8)	.645 [0.10]
		Control	16.1 (0.3)	**-0.8 (-1.4, -0.1)**			**-1.0 (-1.7, -0.4)**		
Micronutrients									
Fibre (g/day)[d]	AI: 14g (aged 3yrs), 18g (aged 4-5 yrs)	Intervention	18.2 (1.7)	-0.8 (-4.1, 2.6)	-2.4 (-6.6, 1.9)	.273 [0.23]	**+3.5 (0.1, 6.8)**	-3.3 (-7.5, -1.0)	.133 [0.32]
		Control	17.2 (1.2)	+1.6 (-1.0, 4.1)			**+6.7 (4.1, 9.3)**		
Sodium (mg/day)[d]	AI: 200-400mg (aged 3yrs), 300-600 (aged 4-5yrs)	Intervention	1364 (104)	**-131 (-342, 80)**	-201 (-467, -65)	.137 [0.31]	+148 (-63, 359)	**-347 (-614, -81)**	**.011 [0.54]**
		Control	1227 (73)	+70 (-91, 232)			**+495 (333, 658)**		
Healthy, nutrient dense core foods (% of total energy intake)									
Core foods	≥85%	Intervention	69.7 (1.8)	**+3.1 (0.6, 5.5)**	+2.3 (-0.9, 5.4)	.155 [0.30]	+1.1 (-1.4, 3.6)	+2.4 (-0.8, 5.5)	.142 [0.31
		Control	72.9 (1.3)	+0.8 (-1.1, 2.7)			-1.3 (-3.2, 0.7)		
Vegetables	-	Intervention	6.0 (0.8)	+0.9 (-0.3, 2.1)	+1.0 (-0.5, 2.6)		+0.4 (-0.8, 1.6)	+0.6 (-0.9, 2.2)	.438 [0.16]

		Control	7.2 (0.6)	-0.2 (-1.1, 0.8)		.186 [0.28]	-0.2 (-1.2, 0.8)		
Fruit	-	Intervention	9.7 (0.9)	+0.8 (-0.7, 2.3)	-0.9 (-2.7, 1.0)	.371 [0.19]	+0.8 (-0.7, 2.3)	+0.5 (-1.4, 2.3)	.637 [0.10]
		Control	9.9 (0.7)	**+1.7 (0.5, 2.9)**			+0.3 (-0.8, 1.5)		
Meats	-	Intervention	7.0 (0.8)	+1.3 (-0.3, 2.9)	+0.9 (-1.0, 2.9)	.357 [0.19]	+0.9 (-0.6, 2.5)	+0.8 (-1.2, 2.7)	.443 [0.16]
		Control	7.7 (0.6)	+0.4 (-0.8, 1.6)			+0.2 (-1.0, 1.4)		
Vegetarian	-	Intervention	2.7 (0.5)	-0.0 (-0.8, 0.7)		.856 [0.04]	+0.4 (-0.4, 1.2)		
protein		Control	3.1 (0.4)	+0.1 (-0.5, 0.7)	+0.1 (-1.0, 0.9)		+0.1 (-0.5, 0.6)	+0.4 (-0.6, 1.3)	.472 [0.15]
sources									
Grains [d]	-	Intervention	19.8 (1.2)	-0.5 (-3.1, 2.1)	-1.2 (-4.4, 2.1)	.481 [0.15]	+1.9 (-0.7, 4.5)	-0.5 (-3.8, 2.7)	.749 [0.07]
		Control	19.9 (0.9)	+0.6 (-1.3, 2.6)			**+2.4 (0.4, 4.4)**		
Breakfast	-	Intervention	9.7 (1.0)	-0.9 (-2.9, 1.1)	-0.6 (-3.1, 1.9)	.649 [0.10]	+0.3 (-1.7, 2.3)	+0.8 (-1.7, 3.3)	.525 [0.13]
cereals		Control	9.3 (0.7)	-0.3 (-1.8, 1.2)			-0.5 (-2.1, 1.0)		
Dairy [d]	-	Intervention	27.1 (1.7)	0.0 (-3.4, 3.3)	+1.9 (-2.3, 6.1)	.368 [0.19]	**-4.3 (-7.6, -0.9)**	-0.3 (-1.8, 5.4)	.879 [0.03]
		Control	27.5 (1.2)	-1.9 (-4.5, 0.6)			**-3.9 (-6.5, -1.4)**		
Energy-dense nutrient-poor (EDNP) foods (% of total energy intake)									
EDNP foods	<1 serve (~5-20%)	Intervention	30.3 (1.8)	**-3.1 (-5.5, -0.6)**	-2.3 (-5.4, 0.9)	.155 [0.30]	**-1.1 (-3.6, 1.4)**	-2.4 (-5.5, 0.8)	.142 [0.31]
		Control	27.1 (1.3)	-0.8 (-2.7, 1.1)			+1.3 (-0.7, 3.2)		
Sugar-	-	Intervention	0.7 (0.2)	+0.2 (-0.2, 0.6)		.586 [0.11]	+0.2 (-0.2, 0.6)		
sweetened		Control	0.6 (0.2)	+0.1 (-0.3, 0.4)	+0.1 (-0.4, 0.6)		-0.1 (-0.4, 0.3)	+0.3 (-0.2, 0.8)	.275 [0.23]
beverages									
Prepacked		Intervention	3.8 (0.6)	**-1.4 (-2.4, -0.3)**	-1.2 (-2.5, 0.1)	.079 [0.37]	**-1.7 (-2.7, -0.6)**	**-2.2 (-3.5, -0.9)**	**.001 [0.70]**
snacks	-	Control	3.0 (0.4)	-0.2 (-1.0, 0.6)			+0.5 (-0.3, 1.4)		
Confectionary	-	Intervention	5.5 (0.7)	-0.7 (-1.7, 0.2)	-0.3 (-1.4, 0.9)	.655 [0.09]	-0.2 (-1.6, 1.1)	+0.3 (-1.4, 2.0)	.693 [0.08]
		Control	4.9 (0.5)	-0.5 (-1.2, 0.2)			-0.6 (-1.6, 0.4)		
Baked	-	Intervention	5.8 (0.6)	-0.9 (-2.0, 0.2)	-0.7 (-2.1, 0.7)	.318 [0.21]	-0.6 (-1.7, 0.5)	-0.6 (-2.0, 0.7)	.353 [0.20]
products		Control	5.1 (0.4)	-0.2 (-1.1, 0.6)			+0.1 (-0.8, 0.9)		
Takeaway [d]	-	Intervention	6.9 (0.7)	+0.2 (-0.9, 1.3)	-0.2 (-1.6, 1.2)	.783 [0.06]	**+1.5 (+0.4, 2.6)**	-0.3 (-1.7, 1.1)	.652 [0.09]
		Control	5.9 (0.5)	+0.4 (-0.5, 1.2)			**+1.8 (1.0, 2.7)**		
Condiments	-	Intervention	2.8 (0.4)	+0.2 (-0.6, 1.0)	+0.2 (-0.9, 1.2)	.754 [0.07]	0.0 (-0.8, 0.8)	+0.3 (-0.7, 1.3)	.579 [0.12]
		Control	3.1 (0.3)	0.0 (-0.6, 0.7)			-0.3 (-0.9, 0.4)		
Fatty meats	-	Intervention	2.0 (0.3)	-0.1 (-0.5, 0.3)	+0.1 (-0.6, 0.4)	.760 [0.06]	+0.2 (-0.4, 0.7)	+0.2 (-0.6, 0.9)	.651 [0.10]
		Control	1.8 (0.2)	0.0 (-0.3, 0.3)			0.0 (-0.5, 0.4)		
Meals *(% of total energy intake)*									

Meals with vegetables f	-	Intervention	4.2 (0.6)	**+1.3 (0.1, 2.5)**	+0.9 (-0.6, 2.4)	.257 [0.24]	+0.4 (-0.8, 1.6)	+0.1 (-1.4, 1.7)	.849 [0.04]
		Control	4.7 (0.5)	+0.4 (-0.5, 1.3)			+0.3 (-0.7, 1.2)		
Meals without vegetables g	-	Intervention	1.6 (0.4)	-0.1 (-0.8, 0.6)	+0.1 (-0.8, 1.0)	.757 [0.06]	+0.2 (-0.5, 0.9)	**+0.5 (-0.4, 1.4)**	**.249 [0.24]**
		Control	1.6 (0.3)	-0.2 (-0.8, 0.3)			-0.3 (-0.9, 0.2)		

Bold denotes a significant difference. *1 kcal = 4.186 kJ.a. 10 week value minus baseline; b. Within-group difference (intervention) minus within-group difference (control); c. 9 month value minus baseline; d. Adjusted for child's age; e. Truncated to account for outliers [5] (>3.29 SD truncated to next highest value plus; f. composite score of all protein meals that were consumed with vegetables, g. composite score of all protein meals that were consumed without vegetables; ARFS; Australian Reccommended Food Score, NRV; Nutrient Reference Value, AMDR; Acceptable Macronutrient Distribution Ranges, AI; Acceptable Intake

Table S5: Change in dietary intake of fathers enrolled in the Healthy Youngsters Healthy Dads (HYHD) RCT (per-protocol) n=91

			**Baseline**	**10 weeks change from baseline (Mean, 95% CI)**			**9 months change from baseline (Mean, 95% CI)**		
**Outcome**	**NRV [2-4,6]**	**Group**	**Mean (SE)**	**Within group[a]**	**Mean difference between groups[b]**	***p*-value [Cohen's *d*]**	**Within group[a]**	**Mean difference between groups[b]**	***p*-value [Cohen's *d*]**
Energy (kJ day)[*, d]	-	Intervention	11021 (595)	**-1392 (-2245, -539)**	**-1642 (-2718, -566)**	**.003 [0.63]**	-662 (-1524, 200)	**-1166 (-2237, -96)**	**.033 [0.45]**
		Control	10327 (414)	+250 (-406, 907)			+504 (-131, 1139)		
Diet Quality Score (max score is 73, higher score = better diet quality)									
Total ARFS	-	Intervention	33.3 (1.6)	+0.3 (-1.9, 2.6)	+0.7 (-2.2, 3.6)	.633 [0.10]	-1.3 (-3.6, 1.0)	+0.4 (-2.4, 3.3)	.768 [0.06]
		Control	34.5 (1.1)	-0.4 (-2.1, 1.4)			**-1.8 (-3.5, -0.1)**		
Macronutrients (% of total energy intake)									
Protein	AMDR: 15-25%	Intervention	17.3 (0.5)	+0.9 (0.2, 2.0)	+0.8 (-0.5, 2.2)	.233 [0.25]	+0.9 (-0.2, 1.9)	+0.8 (-0.5, 2.2)	.226 [0.25]
		Control	17.1 (0.4)	+0.1 (-0.8, 0.9)			0.0 (-0.8, 0.8)		
Carbohydrate	AMDR: 45-65%	Intervention	44.0 (1.3)	+0.6 (-1.4, 2.6)	+0.4 (-2.1, 2.9)	.761 [0.06]	-0.3 (-2.3, 1.8)	+0.5 (-2.0, 3.0)	.692 [0.08]
		Control	44.1 (0.9)	+0.2 (-1.4, 1.7)			-0.8 (-2.3, 0.7)		
Fat	AMDR: 20-35%	Intervention	35.3 (1.0)	-0.6 (-2.4, 1.1)	-0.4 (-2.6, 1.7)	.684 [0.09]	-0.4 (-2.2, 1.3)	-0.5 (-2.7, 1.6)	.628 [0.10]
		Control	35.9 (0.7)	-0.2 (-1.5, 1.1)			+0.1 (-1.2, 1.4)		
Saturated fat	<10%	Intervention	13.6 (0.5)	-0.3 (-1.3, 0.6)	-0.4 (-1.5, 0.8)	.526 [0.13]	-0.3 (-1.3, 0.6)	-0.8 (-1.9, 0.4)	.182 [0.28]
		Control	13.3 (0.3)	0.0 (-0.7, 0.7)			+0.5 (-0.2, 1.2)		
Alcohol	-	Intervention	3.7 (0.7)	**-1.1 (-1.8, -0.5)**	-1.0 (-1.8, -0.1)	.022 [0.49]	-0.3 (-2.0, 1.4)	-1.1 (-3.2, 1.1)	.318 [0.21]
		Control	2.8 (0.5)	-0.2 (-0.7, 0.3)			+0.8 (-0.5, 2.1)		
Micronutrients									
Fibre (g/day)	AI: 30g/day (male aged 19-70y)	Intervention	31.1 (1.9)	-1.1 (-3.6, 1.4)	-2.2 (-5.4, 0.9)	.163 [0.30]	+0.3 (-4.6, 5.2)	-1.6 (-7.7, 4.5)	.604 [0.11]
		Control	32.4 (1.3)	+1.2 (-0.8, 3.1)			+1.9 (-1.8, 5.6)		
Sodium (mg/day)	SDT: <2000mg/day (male aged 19-70y).	Intervention	2378 (143)	**-398 (-207, -588)**	**-421 (-663, -179)**	**.001 [0.74]**	-201 (-815, 412)	-471 (-1239, 297)	.226 [0.26]
		Control	2230 (99)	+24 (-125, 172)			+270 (-193, 732)		
Core foods (% of total energy intake)									
Core foods	≥85%	Intervention	61.3 (2.2)	**+5.8 (2.4, 9.2)**	**+4.7 (0.4, 9.1)**		**+4.8 (1.3, 8.3)**	**+4.7 (0.4, 9.0)**	**.020 [0.45]**

		Control	63.0 (1.6)	+1.1 (-1.6, 3.7)		**.033 [0.45]**	+0.1 (-2.4, 2.7)		
Vegetables	-	Intervention	7.7 (0.8)	+1.2 (-0.1, 2.4)	+0.7 (-0.8, 2.3)	.362 [0.19]	+0.7 (-0.6, 2.0)	+0.3 (-1.3, 1.8)	.749 [0.07]
		Control	8.7 (0.5)	+0.4 (-0.5, 1.4)			+0.4 (-0.5, 1.4)		
Fruit[e]	-	Intervention	6.0 (0.8)	**+1.5 (0.2, 2.9)**	+1.4 (-0.3, 3.1)	.110 [0.34]	+0.4 (-1.0, 1.7)	+0.6 (-1.1, 2.3)	.498 [0.14]
		Control	6.6 (0.5)	+0.1 (-0.9, 1.2)			-0.3 (-1.2, 0.8)		
Meats[e]	-	Intervention	14.3 (1.3)	+1.2 (-1.4, 3.9)	+1.5 (-1.9, 4.7)	.385 [0.18]	+1.0 (-1.6, 3.6)	+1.4 (-1.9, 4.7)	.392 [0.18]
		Control	13.4 (0.9)	-0.2 (-2.2, 1.8)			-0.4 (-2.4, 1.5)		
Vegetarian protein sources	-	Intervention	3.1 (0.6)	0.0 (-0.9, 0.9)	+0.3 (-0.8, 1.4)	.619 [0.10]	-0.4 (-1.3, 0.5)	-0.4 (-1.5, 0.7)	.461 [0.15]
		Control	3.6 (0.4)	-0.3 (-0.9, 0.4)			+0.1 (-0.6, 0.7)		
Grains[e]	-	Intervention	20.4 (1.5)	+0.8 (-2.0, 3.6)	+0.5 (-3.0, 4.0)	.789 [0.06]	**+2.9 (0.1, 5.7)**	**4.7 (1.2, 8.2)**	**.009 [0.56]**
		Control	21.6 (1.1)	+0.4 (-1.8, 2.5)			-1.8 (-3.7, 0.3)		
Breakfast cereals	-	Intervention	5.9 (1.0)	+0.4 (-1.1, 1.9)	+0.1 (-1.8, 2.1)	.888 [0.03]	+1.0 (-0.5, 2.6)	**2.1 (0.1, 4.0)**	**.036 [0.44]**
		Control	7.0 (0.7)	+0.3 (-0.9, 1.4)			-1.0 (-2.2, 0.1)		
Dairy	-	Intervention	9.5 (1.1)	+0.6 (-1.9, 3.1)	-0.1 (-3.3, 3.0)	.929 [0.02]	+0.1 (-2.4, 2.7)	-1.5 (-4.6, 1.7)	.354 [0.19]
		Control	9.2 (0.8)	+0.8 (-1.2, 2.7)			+1.6 (-0.2, 3.5)		
Healthy, nutrient dense core foods (% of total energy intake)									
EDNP foods	<3 serves (~5-20%)	Intervention	38.7 (2.2)	**-5.8 (-9.2, -2.4)**	**-4.7 (-9.1, -0.4)**	**.033 [0.45]**	**-4.8 (-8.3, -1.3)**	**-4.7 (-9.0, -0.4)**	**.033 [0.45]**
		Control	37.0 (1.6)	-1.1 (-3.7, 1.6)			-0.1 (-2.7, 2.4)		
Sugar-sweetened beverages	-	Intervention	2.5 (0.6)	+0.5 (-0.5, 1.5)	+0.5 (-0.8, 1.7)	.446 [0.16]	-0.8 (-1.5, 0.0)	-0.2 (-1.1, 0.7)	.672 [0.09]
		Control	1.7 (0.4)	0.0 (-0.7, 0.8)			**-0.6 (-1.1, 0.0)**		
Prepacked snacks	-	Intervention	3.1 (0.5)	-0.5 (-1.3, 0.3)	-0.7 (-1.7, 0.3)	.190 [0.28]	-0.4 (-1.2, 0.4)	-0.9 (-1.9, 0.2)	.099 [0.35]
		Control	2.5 (0.3)	+0.2 (-0.4, 0.8)			+0.4 (-0.2, 1.1)		
Confectionary	-	Intervention	6.5 (0.9)	**-2.0 (-3.5, -0.6)**	-1.5 (-3.4, 0.3)	.047 [0.34]	**-2.2 (-3.7, -0.7)**	**-2.1 (-3.9, -0.2)**	**.027 [0.47]**
		Control	5.8 (0.6)	-0.5 (-1.6, 0.7)			-0.1 (-1.2, 1.0)		
Baked products	-	Intervention	4.6 (0.7)	-0.2 (-1.4, 1.0)	-0.5 (-2.0, 1.0)	.532 [0.13]	0.0 (-1.2, 1.2)	-0.2 (-1.8, 1.2)	.744 [0.07]
		Control	4.4 (0.5)	+0.3 (-0.6, 1.2)			+0.3 (-0.6, 1.2)		
Takeaway / fast foods	-	Intervention	10.0 (1.3)	-1.0 (-2.9, 0.8)	0.0 (-2.3, 2.4)	.968 [0.01]	-0.7 (-2.4, 0.9)	-0.4 (-2.4, 1.7)	.713 [0.08]
		Control	11.3 (0.9)	-1.1 (-2.4, 0.3)			-0.4 (-1.6, 0.8)		
Condiments	-	Intervention	3.1 (0.5)	-0.9 (-1.8, 0.0)	-1.0 (-2.2, 0.2)	.094 [0.35]	-0.4 (1.3, 0.5)	-0.4 (-1.6, 0.8)	.504 [0.14]
		Control	3.4 (0.4)	0.1 (-0.6, 0.8)			0.0 (-0.7, 0.7)		
Fatty meats	-	Intervention	2.8 (0.3)	0.0 (-0.5, 0.6)	-0.2 (-0.5, 0.9)	.588 [0.11]	-0.0 (-0.6, 0.6)	0.6 (-0.1, 1.3)	.107 [0.34]
		Control	2.7 (0.2)	-0.1 (-0.5, 0.3)			**-0.6 (-1.0, -0.2)**		
Meals *(% of total energy intake)*									

Meals with vegetables (%)[f]	-	Intervention	8.9 (0.9)	+1.0 (-0.9, 2.8)	1.2 (-1.1, 3.5)	.320 [0.21]	+0.9 (-1.0, 2.7)	+1.4 (-0.9, 3.7)	.242 [0.25]
		Control	8.7 (0.6)	-0.2 (-1.6, 1.2)			-0.6 (-1.9, 0.8)		
Meals without vegetables (%)[g]	-	Intervention	3.0 (0.7)	-0.0 (-1.4, 1.3)	-0.1 (-1.8, 1.6)	.910 [0.02]	-0.1 (-1.3, 1.4)	0.0 (-1.7, 1.7)	.997 [0.00]
		Control	2.5 (0.5)	+0.1 (-1.0, 1.1)			+0.1 (-0.9, 1.1)		

Bold denotes a significant difference. *1 kcal = 4.186 kJ.a. 10 week value minus baseline; b. Within-group difference (intervention) minus within-group difference (control); c. 9 month value minus baseline; d.Truncated to account for outliers [5] (>3.29 SD truncated to next highest value plus 1) e. Adjusted for father's age; f. composite score of all protein meals that were consumed with vegetables, g. composite score of all protein meals that were consumed without vegetables; ARFS; Australian Reccommended Food Score, NRV; Nutirent Reference Value, AMDR; Acceptable Macronutrient Distribution Ranges, AI; Acceptable Intake, SDT: Suggested Dietary Target

1. Eat for Health. Recommended number of serves for children, adolescents and toddlers. Availabe online: https://www.eatforhealth.gov.au/food-essentials/how-much-do-we-need-each-day/recommended-number-serves-children-adolescents-and (accessed on May 25th).
2. National Health and Medical Research Council. Nutrient Reference Values for Australia and New Zealand: Macronutrient balance. Availabe online: https://www.nrv.gov.au/chronic-disease/macronutrient-balance (accessed on May 25th).
3. National Health and Medical Research Council. Nutrient Reference Values for Australia and New Zealand: Sodium. Availabe online: https://www.nrv.gov.au/nutrients/sodium (accessed on May 25th).
4. National Health and Medical Research Council. Nutrient Reference Values for Australia and New Zealand: Dietary Fibre Availabe online: https://www.nrv.gov.au/nutrients/dietary-fibre (accessed on May 25th).
5. Tabachnic BG FL. *Using Multivariate Statistic. 3rd ed.* ; Allyn & Bacon: Boston, MA:, 2001.
6. Eat for Health. Recommended number of serves for adults. Availabe online: https://www.eatforhealth.gov.au/food-essentials/how-much-do-we-need-each-day/recommended-number-serves-adults (accessed on May 25th).
